# Medial vs Central Upper Eyelid Fat: A Histological and Immunohistochemical Study of Structural and Vascular Features

**DOI:** 10.1093/asjof/ojag149

**Published:** 2026-07-14

**Authors:** Lukas Prantl, Hanna Guice, Sophia-Theresa Diesch, Andreas Eigenberger, Christina Gottwald, Ana Cristina S R G Jorge, Rafael Loucas, Andrea Pagani, Kirsten Utpatel, Tom Schimanski

## Abstract

**Background:**

The medial upper eyelid fat compartment has long been recognized clinically as being firmer, whiter, and more fibrous than the central preaponeurotic fat. Despite its surgical relevance, systematic quantitative histological characterization remains limited.

**Objectives:**

The objective of this study was to quantitatively compare the histological and immunohistochemical characteristics of medial and central upper eyelid fat compartments, with particular focus on connective tissue composition, vascularization, and stromal nuclear density.

**Methods:**

In a paired-sample design, medial and central upper eyelid fat compartments were harvested from 10 patients undergoing upper blepharoplasty. Each compartment was analyzed using hematoxylin and eosin (H&E) staining, acid fuchsin + orange G staining, and CD31 immunohistochemistry. Connective tissue proportion, capillary density, and stromal nuclear density were assessed.

**Results:**

The medial compartment demonstrated a significantly higher proportion of connective tissue compared with the central compartment (67.74 ± 5.63% vs 56.63 ± 5.56%, *P* < .001). In contrast, capillary density was significantly reduced in the medial compartment (1.60 ± 0.54% vs 3.04 ± 1.14%, *P* = .002). Stromal nuclear density was also lower in medial fat compared with central fat (0.85 ± 0.66% vs 1.76 ± 1.06%, *P* = .004). Across all parameters, the central compartment consistently showed higher stromal nuclear density and vascularization, whereas the medial fat exhibited increased fibrous density.

**Conclusions:**

The medial upper eyelid fat compartment differs histologically from the central compartment by an increased connective tissue content and reduced vascular and stromal cell density. These findings provide a structural basis for its clinically observed firmness and potentially altered regenerative behavior.

The upper eyelid contains multiple anatomically distinct fat compartments that contribute significantly to both eyelid function and aesthetic appearance.^1,2^ These compartments not only provide volume and contour but also influence eyelid mobility and biomechanical behavior.^3,4^ In clinical practice, the medial (nasal) upper eyelid fat compartment is consistently perceived as structurally different from the central preaponeurotic fat. Surgeons frequently describe the medial fat pad as paler, firmer, and more fibrous in texture, often appearing more clearly encapsulated than adjacent adipose tissue.^4,5^

These macroscopic differences have practical implications. During blepharoplasty, preservation or repositioning of upper eyelid fat has become increasingly important to avoid postoperative hollowing and to maintain a natural eyelid contour.^6-8^ However, despite its routine surgical handling, the structural properties of those compartments remain insufficiently characterized at the microscopic level. Although orbital adipose tissue has been described anatomically as a compartmentalized and septated structure with supportive fibrous elements and intrinsic vascular networks, systematic quantitative comparisons between medial and central upper eyelid fat compartments are scarce.^9^

Most available descriptions remain qualitative and focus primarily on gross anatomy and comparison to subcutaneous fat rather than morphometric analysis and comparison of different fat compartments in the upper eyelid.^10,11^ Yet structural differences at the histological level, such as variations in connective tissue density, capillary distribution, or adipocyte morphology, may directly influence mechanical behavior, graft integration, and tissue remodeling after surgical manipulation. Increased connective tissue content could explain the clinically observed firmness of medial fat pads, whereas reduced vascular density might account for their paler appearance.

Moreover, vascularization is a key determinant of tissue integration and remodeling following transposition.^12-14^ However, tissue survival after grafting is also influenced by recipient-site vascular ingrowth and other local biological factors. Therefore, intrinsic vascular differences between compartments should be interpreted cautiously and cannot directly predict clinical graft viability.

Against this background, a standardized and reproducible histological workflow is essential to objectively validate clinical observations. Quantitative morphometric analysis, including color deconvolution-based segmentation and paired intra-individual comparison, offers a robust approach to translate empirical surgical impressions into measurable structural parameters.

Therefore, the aim of the present study was to perform a detailed histological and immunohistochemical comparison of medial and central upper eyelid fat compartments. Using hematoxylin and eosin (H&E) staining, acid fuchsin + orange G (SFOG) trichrome staining, and CD31 immunohistochemistry in a paired-sample design, we quantified connective tissue proportion, vascular density, and stromal nuclear density within each compartment. By directly comparing medial and central fat pads within the same individuals, we sought to minimize inter-individual variability and provide a precise structural characterization of these clinically relevant adipose depots.

## METHODS

### Patient Selection and Surgical Procedure

This study was conducted in accordance with the principles of the Declaration of Helsinki and approved by the local institutional Ethics Committee of the University Hospital of Regensburg (24-3640-101). Our samples were obtained from 10 patients (6 female and 4 male) with a mean age of 67.2 ± 9.1 years and a mean BMI of 24.2 ± 2.5 kg/m^2^. Patients were included using a consecutive sampling approach from all eligible upper blepharoplasty cases between August 2025 and March 2026. Inclusion criteria comprised availability of both medial and central upper eyelid fat compartments for histological analysis and absence of previous periocular or orbital surgery. All patients meeting these criteria and providing written consent before the procedure were included.

Elective upper eyelid surgery was performed under uncomplicated general anesthesia in each case. Intraoperative assessment determined whether the upper eyelid fat pads would be preserved or resected, based on clinical and aesthetic considerations. Only patients who had the indication for medial and central fat compartment resection were included in this study. For each included individual, 4 distinct fat samples were collected: medial and central fat pads from both the right and left upper eyelids, allowing intra-individual comparisons while controlling for inter-patient variability.

### Tissue Processing and Histological Preparation

Excised tissue samples were fixed in neutral buffered formalin and embedded in paraffin. Paraffin blocks were sectioned at a thickness of 4 µm. Sections were deparaffinized in xylene and rehydrated through a graded series of alcohols before staining. H&E (Merck KGaA, Darmstadt, Germany) staining was performed to assess general morphology and adipocyte architecture. Additional sections were subjected to SFOG (Merck KGaA) staining to highlight connective tissue structures and to immunohistochemical staining for CD31 (Clone JC70A; Dako Omnis, Agilent Technologies, Santa Clara, CA) to visualize endothelial cells and vascular structures. All stained slides were digitized using a P1000 whole-slide scanner (3D Histech Kft., Budapest, Hungary) at high resolution, ensuring consistent image quality for quantitative analysis.

### Image Calibration and Standardization

To ensure reproducibility and comparability across samples, all digital images were calibrated using scale bars present in the exported slides. Images were processed and analyzed using FIJI/ImageJ (Version 20250529-2217, National Institutes of Health, Bethesda, MD), a widely adopted open-source image analysis platform that supports standardized workflows across multiple staining modalities and enables reproducible quantification of biological structures. Consistent magnification was maintained during image export from the slide viewer, and all subsequent analysis preserved this calibration to allow standardized pixel size. Artifacts such as folds, tears, and staining irregularities were minimized through careful manual inspection from 2 independent investigators (H.G. and T.S.) and excluded from analysis wherever necessary.

### Quantitative Image Analysis

#### Connective Tissue Quantification

SFOG-stained images were imported into FIJI/ImageJ for morphometric evaluation. Color deconvolution was applied to separate staining components, and the blue channel, representing collagen-associated structures, was selected for further analysis. Binary masks were generated using standardized thresholding to identify all blue-stained regions. Importantly, this approach captures not only interstitial collagen but also collagen-rich structures associated with adipocyte boundaries, including pericellular matrix and membrane. Rather than attempting to artificially separate these closely related structures, all blue-stained elements were consistently quantified as a composite compartment. This strategy minimizes subjective bias introduced by manual editing and ensures high reproducibility across samples by relying on a uniform, threshold-based segmentation approach. The total tissue area was defined by excluding background and nontissue regions. Connective tissue content was then expressed as the proportion of blue-stained area relative to the total tissue area. To ensure methodological robustness, threshold settings were standardized across all images and visually validated. Particular care was taken to confirm that adipocyte cytoplasm was not misclassified as connective tissue during segmentation. Accordingly, the reported values reflect the fraction of collagen-associated and membrane-adjacent structures within the tissue, rather than exclusively interstitial connective tissue in the narrow histological sense.

#### Vascularization Analysis

Capillary structures were quantified from CD31 immunohistochemical sections. Color deconvolution using the H DAB algorithm was used to separate the brown DAB signal representing CD31-positive endothelial cells. Binary masks were created from both the DAB and hematoxylin channels, and background was removed to isolate relevant tissue. The area of CD31-positive staining was quantified relative to the total tissue area for each section, allowing assessment of vascularization as both absolute area and percentage of tissue.

#### Quantification of Stromal Nuclear Density

To further characterize compartment-specific differences in regenerative potential, stromal nuclear density was quantified on H&E-stained sections.

Importantly, this analysis does not represent a direct characterization of stromal vascular fraction (SVF) or its cellular subpopulations. Because no immunophenotypic staining or viability-based assessment was performed, the quantified parameter should be interpreted as a morphometric estimate of nonadipocyte stromal nuclear density within adipose tissue regions rather than a specific measurement of SVF cells. A precise characterization of SVF components would require dedicated immunophenotypic analysis using established marker panels.

From each histological image, a standardized region of interest (ROI) was selected at 8-fold digital magnification.

Although higher magnifications (eg, 10×, 20×, or 40×) are more commonly used, 8× was deliberately chosen as it provided an optimal balance between field size and sufficient resolution to reliably detect and quantify all nuclei within adipose tissue while maintaining a representative overview of the tissue architecture.

Within each ROI, all visible nuclei were quantified. Given the exclusive selection of regions containing predominantly adipose tissue with minimal connective tissue components, it was assumed that the majority of counted nuclei correspond to cells of the SVF, including adipose-derived stromal/stem cells.

To ensure methodological consistency, ROI selection followed predefined criteria (adipose tissue only, minimal fibrous septa), and identical analysis parameters were applied across all samples. Quantification was performed by one observer using the same protocol.

Within the ROI, the hematoxylin (blue) channel was isolated using color deconvolution. Nuclear profiles consistent with stromal cell morphology (small, elongated to oval nuclei located within the interstitial space between adipocytes) were identified and quantified using semi-automated image analysis.

### Statistical Analysis

Quantitative results are reported as mean ± standard deviation unless otherwise noted. Normality of all measured parameters was assessed using the Kolmogorov–Smirnov test separately for medial and central compartments across all variables. None of the tested parameters showed a significant deviation from a normal distribution (*P* > .05), confirming that the assumption of normality was met for all subsequent parametric analyses. Paired Student's *t*-tests were applied to compare morphometric measures between medial and central fat compartments within the same individuals, thereby reducing inter-individual variability and increasing analytical sensitivity. To account for multiple testing across 3 endpoints, *P*-values were adjusted using the Holm–Bonferroni method. In addition to *P*-values, effect sizes were calculated using Cohen's *d* for paired samples. The level of statistical significance was set at *α* = .05. All statistical analyses were performed using Microsoft Excel (Microsoft 365/Excel 2024, Microsoft Corporation, Redmond, WA).

## RESULTS

### Patient Demographics

Ten patients (6 female, 4 male) with a mean age of 67.2 ± 9.1 years and a mean BMI of 24.2 ± 2.5 kg/m^2^ were included in this study. All included patients originated from an ethnically homogeneous Central European population treated at our institution. No previous periocular or orbital surgery was present in any case.

### General Histological Appearance

H&E staining demonstrated well-preserved adipocyte architecture in both compartments, with visible septal structures separating adipocyte clusters. SFOG staining highlighted collagen-rich connective tissue structures, which appeared more pronounced and more densely organized in medial fat compartments. CD31 immunohistochemistry demonstrated endothelial-lined capillaries throughout all specimens, with visually reduced vascular density in medial compartments compared with central compartments.

**Figure 1.**
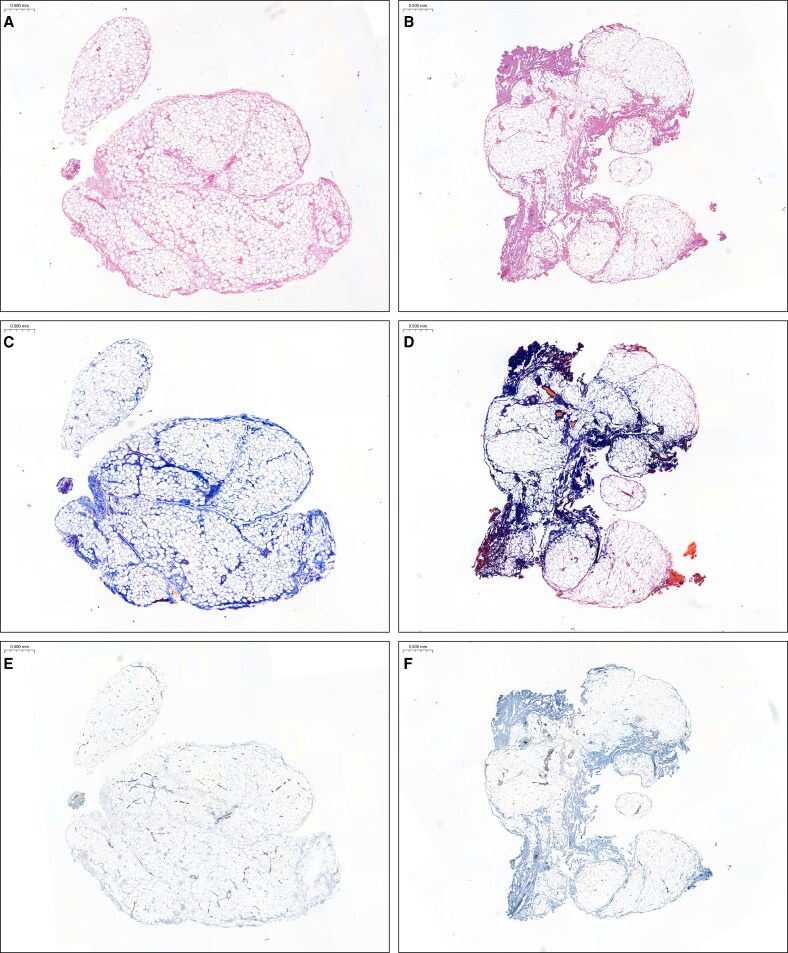
Representative hematoxylin and eosin (H&E), acid fuchsin + orange G (SFOG), and CD31 stainings of the central (left) and medial (right) upper eyelid fat compartments, obtained from the same patient and the same side, each shown at 2-fold magnification. (A) central fat compartment, H&E staining, (B) medial fat compartment, H&E staining, (C) central fat compartment, SFOG staining, (D) medial fat compartment, SFOG staining, (E) central fat compartment, CD31 immunohistochemistry, (F) medial fat compartment, CD31 immunohistochemistry. In H&E staining, differences are primarily reflected in nuclear density and distribution, with the medial compartment showing a comparatively higher stromal cell/nuclear density within the adipose tissue. In SFOG staining, the medial compartment demonstrates increased connective tissue with more prominent fibrous septa and extracellular matrix deposition compared with the central compartment. In CD31 immunohistochemistry, the central compartment shows a higher density of CD31-positive microvessels with a more continuous and evenly distributed capillary network, whereas the medial compartment exhibits reduced vascular density.

An overview of representative stainings, including whole-section images of central and medial fat compartments, is shown in Figure 1.

### Quantitative Connective Tissue Analysis

**Figure 2.**
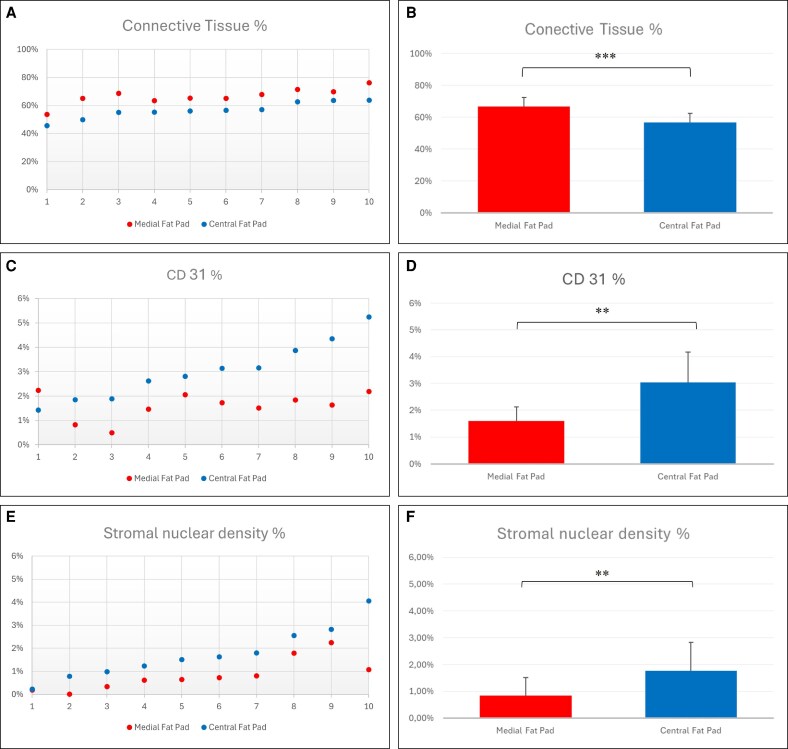
Comparison of medial and central upper eyelid fat pads. Left panels: individual data points for connective tissue fraction (% connective tissue), CD31-positive area (CD31%), and stromal nuclear density (%) in medial (red) and central (blue) fat pads. Right panels: mean ± standard deviation with significance indicators (**P < .01; ***P < .001). (A) Individual connective tissue fraction (%) per patient in medial (red) and central (blue) fat pads. (B) Mean connective tissue fraction (%) with group comparison and significance analysis. (C) Individual CD31-positive area (%) per patient in medial and central fat compartments. (D) Mean CD31-positive area (%) with group comparison and significance analysis. (E) Individual stromal nuclear density (%) per patient in medial and central fat pads. (F) Mean stromal nuclear density (%) with group comparison and significance analysis. The central compartment demonstrated consistently higher cellular and vascular parameters, whereas the medial compartment showed increased connective tissue proportion.

Quantitative morphometric analysis of SFOG-stained sections demonstrated a significantly higher proportion of connective tissue in medial fat compartments compared with central compartments as can be seen in Figure 2. The mean connective tissue content in medial fat pads was 67.74 ± 5.63%, whereas central compartments showed a mean proportion of 56.63 ± 5.56%. The effect size for paired comparisons, calculated as Cohen's d, was 3.74, indicating a very large effect. This difference reached statistical significance (paired t-test, *P* < .001). Normality testing using the Kolmogorov–Smirnov test confirmed normal distribution for this parameter (medial: *P* = .20, central: *P* = .22).

### Quantitative Vascularization Analysis

Analysis of CD31-stained sections revealed a significantly lower proportion of CD31-positive area in medial fat compartments compared with central compartments. The mean vascularized area in central compartments was 3.04 ± 1.14%, whereas medial compartments demonstrated a mean vascular area of 1.60 ± 0.54%. The effect size for paired comparisons, calculated as Cohen's d, was 1.41, indicating a very large effect. The difference was statistically significant (paired t-test, *P* = .002). Normality testing using the Kolmogorov–Smirnov test confirmed normal distribution for this parameter (medial: *P* = .21, central: *P* = .33).

**Figure 3.**
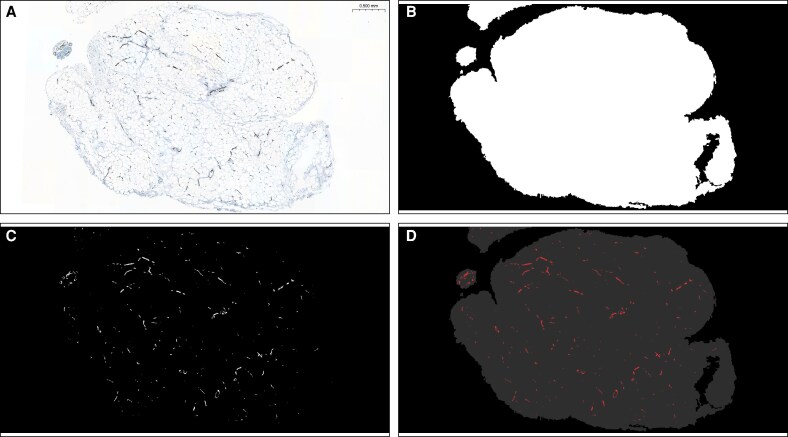
(A) Representative CD31 immunohistochemical staining of a central upper eyelid fat compartment. (B) Binary mask of the total tissue area obtained after color deconvolution and thresholding. (C) Binary mask of CD31-positive structures within the same region of interest. (D) Overlay of the total tissue area (B) and CD31-positive area (C), included for visual illustration purposes only and not used for quantitative analysis. Quantitative measurements were derived exclusively from the independently generated masks in B and C, allowing calculation of CD31-positive area relative to the total tissue area.

A representative example of the image analysis workflow for vascular quantification in 1 patient (left medial fat compartment) is shown in Figure 3, illustrating the original CD31 staining, color deconvolution, binary mask generation, and area calculation.

### Stromal Cell Density

Quantitative analysis revealed a higher stromal nuclear density in the central compartment compared with the medial compartment. The mean density in medial compartments was 0.85 ± 0.66%, whereas central compartments demonstrated a density of 1.76 ± 1.06%. The effect size for paired comparisons, calculated as Cohen's d, was 1.25, indicating a very large effect. The difference was statistically significant (paired t-test, *P* = 4.45 × 10^−3^). Normality testing using the Kolmogorov–Smirnov test confirmed normal distribution for this parameter (medial: *P* = .28, central: *P* = .32).

## DISCUSSION

The present study provides a quantitative histological and immunohistochemical comparison of medial and central upper eyelid fat compartments. Our findings demonstrate that medial fat pads contain a significantly higher proportion of connective tissue and exhibit significantly reduced vascularization compared with central compartments. The comparatively high proportion of blue-stained structures can be partly explained by geometric considerations. In line with principles described by Bergmann's rule, smaller structural units exhibit an increased surface-to-volume ratio, resulting in a relatively greater contribution of boundary-associated, collagen-rich components.^15^ This effect is particularly relevant in the investigated adipose compartments and further contributes to the elevated measured fractions. Consequently, these values should not be interpreted as equivalent to classical interstitial connective tissue content but rather as a composite measure of collagen-associated structures. Furthermore, normalization of vascular area to connective tissue content revealed a markedly lower capillary-to-stroma ratio in medial fat pads, indicating a relative hypovascularization beyond mere stromal expansion.

Orbital adipose tissue has traditionally been described as a specialized fat depot with both structural and functional characteristics distinct from subcutaneous fat.^16-18^ Orbital adipose tissue differs structurally and functionally from subcutaneous adipose tissue, acting as a protective and supportive cushion within the orbit while also contributing to ocular motility and the positioning of the globe.^19,20^ It is described as compartmentalized, with fibrous septa providing structural organization and contributing to mechanical stability within the orbit.^2,21^

However, although macroscopic and anatomical compartmentalization is well recognized, quantitative histological comparisons between medial and central upper eyelid fat compartments have remained limited. Our findings refine the anatomical understanding by demonstrating that the medial fat compartment is not only macroscopically distinct but also microscopically characterized by increased connective tissue density and reduced vascularization.

Adipose-derived stromal cells are considered key mediators of tissue regeneration, angiogenesis, and extracellular matrix remodeling.^22-25^ A lower stromal cell density, in combination with reduced vascularization and increased connective tissue content, in theory limits the regenerative and adaptive capacity of the medial fat body following surgical manipulation. Importantly, stromal nuclear density represents a histomorphometric estimate based on nuclear quantification in adipose tissue regions and does not constitute a direct measurement of isolated SVF cells.

**Figure 4.**
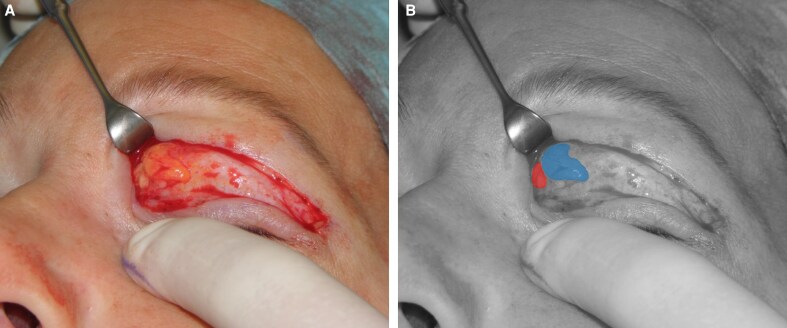
Intraoperative view during blepharoplasty on a 46-year-old woman. (A) Native intraoperative appearance of the upper eyelid fat pads. (B) Color-coded overlay of the fat compartments, with the central fat compartment highlighted in blue and the medial fat compartment highlighted in red. The paler and deeper location of the medial fat compartment within the orbit is noticeable.

In clinical practice, surgeons frequently observe that the medial fat compartment appears paler and firmer than the central fat pad, as can be seen in Figure 4.^26^ The present data provide a histological explanation for this long-standing empirical impression. The increased connective tissue content likely contributes to the firmer consistency, whereas the reduced vascular density plausibly explains the lighter appearance observed intraoperatively.

Historical applications of orbital fat transposition and vascularized fat flaps have been described in both lower and upper eyelid surgery. Goldberg introduced transconjunctival orbital fat repositioning for lower eyelid rejuvenation, whereas Yoo et al later described the use of transposed upper eyelid fat for correction of orbitoglabellar deformities.^27,28^

Therefore, a detailed structural characterization of distinct upper eyelid fat compartments is clinically relevant for improving the anatomical understanding of compartment-specific tissue properties in periocular surgery.

However, the present study was not designed to evaluate clinical graft integration, perfusion, viability, or surgical outcomes following fat repositioning procedures. In particular, the CD31-positive vascular area quantified in this study represents a structural histological parameter and should not be interpreted as a direct surrogate for functional perfusion pressure or tissue viability in vivo. Therefore, the observed histological differences between medial and central upper eyelid fat compartments should not be interpreted as supporting or discouraging specific surgical techniques.

Future in vivo investigations will be necessary to determine whether the structural differences observed in this study translate into clinically relevant differences following transplantation or repositioning procedures.

Beyond the anatomical findings, this study serves as a proof of principle demonstrating how clinical observations can be systematically translated into quantitative histological investigation. The workflow implemented here, including standardized fixation, defined staining protocols, digital whole-slide imaging, reproducible color deconvolution, mask-based quantification, and paired statistical analysis, provides a transparent and replicable framework.

Importantly, the methodological approach was guided by previously formulated recommendations for rigorous histological analysis, as outlined in our previous reviews on adipose tissue histology and quality control.^29-33^ By adhering to predefined analytical principles, including standardized ROI definition, artifact exclusion, and compartment-specific paired comparison, this study illustrates how reproducible histological research can bridge clinical hypotheses and structural validation.

Several limitations must be acknowledged. Histological processing inherently introduces potential artifacts, including tissue shrinkage, sectioning distortion, and staining variability. Although careful manual correction and standardized processing were applied, residual artifacts cannot be fully excluded.

SVF cell quantification was based on morphological identification of nuclei within adipose tissue on H&E-stained sections. Although this approach is commonly used in histomorphometric analyses, it does not allow definitive identification of SVF subpopulations and should therefore be interpreted as an estimate of cellular density rather than a direct measurement of SVF composition. We therefore used the phrase stromal nuclear density instead of SVF throughout this manuscript.

Furthermore, histological analysis is time-consuming and resource intensive, limiting the achievable sample size. Although the inclusion of 10 patients appear limited, the paired design yielded 4 fat samples per patient (bilateral medial and central compartments), resulting in a total of 40 fat depots analyzed. Each depot was evaluated in 3 different staining modalities. For a proof-of-principle morphometric investigation, the paired intra-individual design allows robust detection of consistent structural differences while minimizing inter-individual variability.

Another limitation of the present study is that no functional or in vivo assessment of graft integration, perfusion, or tissue viability was performed. Consequently, the observed histological differences between medial and central fat compartments cannot be directly translated into clinical behavior following transplantation or repositioning procedures.

To further validate the functional implications of our findings, future studies should incorporate in vivo experimental models. Transplantation of medial and central fat pads could allow direct comparison of integration, fibrosis formation, and revascularization capacity. Such experiments would clarify whether the histologically observed differences translate into measurable differences in graft survival, mechanical properties, or fibrotic remodeling after transplantation.

Although reduced intrinsic vascular density was observed in the medial compartment, the functional relevance for graft survival remains uncertain, because tissue viability after transposition is also dependent on recipient-site vascular ingrowth and local healing conditions.

Importantly, this work illustrates how clinical impressions can be systematically translated into reproducible histological analysis. Although limited by sample size and inherent histological constraints, the paired-compartment design and multi-parameter evaluation provide a robust proof-of-principle framework. Future studies incorporating in vivo transplantation models will be required to determine whether the medial fat compartment exhibits altered integration dynamics or fibrosis susceptibility following repositioning.

## CONCLUSIONS

This study provides quantitative histological evidence that the medial upper eyelid fat compartment is structurally distinct from the central preaponeurotic fat. Increased connective tissue-associated staining, reduced capillary density, and lower morphometrically estimated stromal nuclear density characterize the medial fat body and likely contribute to its clinically observed firmness and lighter appearance.
